# Nutrigenetic Interaction Between Apolipoprotein C3 Polymorphism and Fat Intake in People with Nonalcoholic Fatty Liver Disease

**DOI:** 10.1016/j.cdnut.2023.100051

**Published:** 2023-03-02

**Authors:** Reina Yamamoto, Yumie Takeshita, Hiromasa Tsujiguchi, Takayuki Kannon, Takehiro Sato, Kazuyoshi Hosomichi, Keita Suzuki, Yuki Kita, Takeo Tanaka, Hisanori Goto, Yujiro Nakano, Tatsuya Yamashita, Shuichi Kaneko, Atsushi Tajima, Hiroyuki Nakamura, Toshinari Takamura

**Affiliations:** 1 Department of Endocrinology and Metabolism, Kanazawa University Graduate School of Medical Sciences, Kanazawa, Japan; 2 Department of Environmental and Preventive Medicine, Kanazawa University Graduate School of Medical Sciences, Kanazawa, Japan; 3 Department of Bioinformatics and Genomics, Kanazawa University Graduate School of Medical Sciences, Kanazawa, Japan; 4 Department of Gastroenterology, Kanazawa University Graduate School of Medical Sciences, Kanazawa, Japan

**Keywords:** nonalcoholic fatty liver disease, polymorphism, apolipoprotein C3, fat intake, carbohydrate intake, BDHQ, lipoprotein lipase

## Abstract

**Background:**

Recent genome-wide association studies have revealed that nonalcoholic fatty liver disease (NAFLD) is correlated with genetic polymorphisms. However, the effects of genetic variation on nutritional metabolism and NAFLD are complex and further studies are still needed.

**Objectives:**

This study aimed to assess the nutritional characteristics interacting with the correlation between genetic predisposition and NAFLD.

**Methods:**

We assessed the 2013–2017 health examination data of 1191 adults aged ≥40 y living in Shika town, Ishikawa Prefecture, Japan. Adults with moderate or heavy alcohol consumption and hepatitis were excluded, and 464 participants who underwent genetic analyses were included in the study. Abdominal echography was performed to diagnose fatty liver condition, and dietary intake and nutritional balance were evaluated using the brief self-administered diet history questionnaire. NAFLD-related gene polymorphisms were identified using Japonica Array v2 (Toshiba).

**Results:**

Among the 31 single nucleotide polymorphisms, only the polymorphism T-455C in the apolipoprotein C3 (*APOC3*) gene (rs2854116) was significantly associated with fatty liver condition. The condition was more common in participants with heterozygotes of the *APOC3* gene (rs2854116) than in those with the TT and CC genotypes. Significant interactions were observed between NAFLD and the intake of fat, vegetable fat, MUFAs, PUFAs, cholesterol, n-3 FAs, and n-6 FAs. Moreover, participants with NAFLD who presented with the TT genotype had a significantly higher fat intake than those without NAFLD.

**Conclusions:**

The polymorphism T-455C in the *APOC3* gene (rs2854116) and fat intake are associated with the NAFLD risk in Japanese adults. Participants with a fatty liver who presented with the TT genotype of rs2854116 had a higher fat intake. Such nutrigenetic interaction can deepen our understanding of the NAFLD pathology. Moreover, in clinical settings, the correlation between genetic factors and nutrition intake should be considered in personalized nutritional interventions against NAFLD. *Curr Dev Nutr* 2023;xx:xx.

The study was registered in the University Hospital Medical Information Network Clinical Trials Registry as UMIN 000024915.

## Introduction

Nonalcoholic fatty liver disease (NAFLD) is the most common chronic liver condition worldwide, affecting 25% of the adult population. It is classified into 2 types: simple fatty liver and nonalcoholic steatohepatitis (NASH). In some cases, NASH progresses into cirrhosis and liver cancer [1]. Even in the Japanese population, which is considered to have a relatively high percentage of nonobese people, the prevalence of NAFLD is as high as 29.7% and is continually increasing with the increasing obese population [2]. However, the effect of such racial differences on the NAFLD pathology remains unexplained.

Both genetic predisposition and environmental factors contribute to NAFLD development [3]. Genome-wide association studies revealed that several risk alleles of genes such as *PNPLA3* and *GCKR* are associated with the prevalence of NAFLD/NASH [[4], [5], [6]]. Candidate gene studies also identified various other SNPs associated with NAFLD. One study in the United States has revealed an association between apolipoprotein C3 (*APOC3*) mutations and NAFLD. In nonobese Asian Indian men, carriers of the T allele of *APOC3* C-482T (rs2854117), the C allele of *APOC3* T-455C (rs2854116), or both are associated with elevated APOC3 concentrations, reduced plasma TG clearance, hypertriglyceridemia, and a higher prevalence of NAFLD compared with the homozygotes of the opposite alleles [7]. APOC3 is predominantly produced in the liver and functions as a protein inhibitor of LPL. It suppresses TG hydrolysis and reduces TG clearance, leading to elevated TG concentrations [8,9]. Hepatic APOC3 expression is induced by glucose, fructose [10], and SFAs [11] and downregulated by insulin [12] and PUFAs [13]. The *APOC3* gene T-455C variant is located in the insulin response element in the promoter region and causes the loss of insulin regulation, leading to *APOC3* overexpression [14]. Because APOC3 is an LPL inhibitor, the insulin-independent upregulation of APOC3 inhibits LPL, which reduces the hydrolysis of chylomicron, leading to hypertriglyceridemia [7]. Conversely, *APOC3* T-455C–associated LPL dysfunction impairs lipolysis. APOC3 is also overexpressed in type 1 and type 2 diabetes and is associated with relative insulin deficiency or insulin resistance [15].

In addition to these genetic backgrounds, pathophysiological and nutritional factors, including insulin resistance, diabetes, obesity, metabolic syndrome, and excessive fat or carbohydrate intake, are closely correlated with the pathological conditions of NAFLD [16]. Several studies have demonstrated the interaction between NAFLD and single nucleotide polymorphisms (SNPs) and dietary habits [[17], [18], [19]]. For example, Hispanic children with a GG genotype of the *PNPLA3* I148M gene (rs738409 C > G) who had a high carbohydrate intake, particularly total sugar, presented with a high hepatic fat fraction [17]. *PNPLA3* I148M polymorphism was more strongly associated with steatosis in people who consume sweet drinks. In addition, the association between *PNPLA3* I148M polymorphism and steatosis severity was weaker in individuals with a poor vegetable diet [18]. However, the effects of genetic variation on nutrient metabolism and the susceptibility to NAFLD are complex, and research is still needed on the impact of genetic variations on nutrient-related pathways and on how they are affected by aspects of nutrition. Specifically, the association between SNPs other than *PNPLA3* and nutrients is unknown and has not been reported for Asians. In addition, these findings have been insufficiently applied for personalized nutritional interventions against NAFLD in clinical settings. Nutrigenomics, a branch of science that investigates the interaction between nutrition and genetic factors, could be better explored [20]. A deeper understanding of the interaction between genomics and diet may lead to the identification of noninvasive biomarkers representative of the human nutritional status.

This study investigated the nutritional characteristics interacting with the correlation between genetic predisposition and NAFLD in a general population in Japan.

## Methods

### Study population

This cross-sectional study was based on the Shika study, an ongoing population-based survey in Shika town, located in the Ishikawa Prefecture, with a population of ∼20,000. Data were obtained via a survey using a self-administered questionnaire and comprehensive health examination performed between 2013 and 2017.

This study was performed in accordance with the principles of the Declaration of Helsinki, and the investigational protocol was approved by the Ethics Committee for Human Studies at Kanazawa University Hospital (1491, 326). The study was registered in the University Hospital Medical Information Network (UMIN) Clinical Trials Registry as UMIN 000024915. All patients provided written informed consent.

**FIGURE 1:**
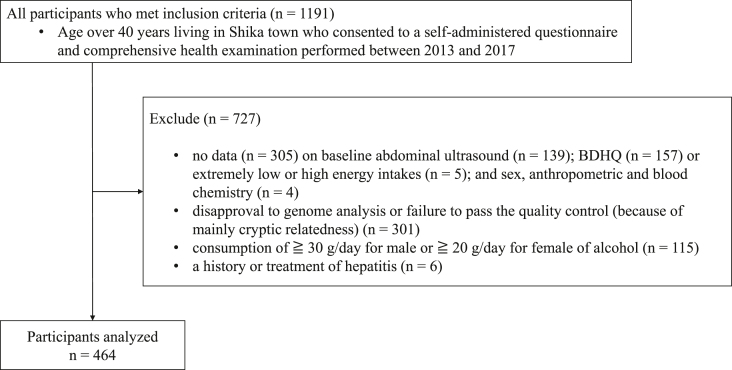
Study design and flowchart of study participants. BDHQ, brief self-administered diet history questionnaire.

Figure 1 shows the study design and enrollment procedure. A total of 1191 adult participants aged ≥40 who lived in rural areas were enrolled in this research. Among them, 727 participants were excluded because of the following reasons: disapproval of genome analysis or failure to pass the quality control (QC) mainly because of cryptic relatedness (*n* = 301, as described in the subsection Genetic analyses); extremely low or high EIs (*n* = 5); history or treatment of hepatitis (*n* = 6); alcohol consumption of ≥30 g/d for men or ≥20 g/d for women (*n* = 115); lack of data about sex, anthropometric, and blood chemistry (*n* = 4); baseline abdominal ultrasonography results (*n* = 139); and brief self-administered diet history questionnaire (BDHQ) findings to assess nutrient intake (*n* = 157). Finally, 464 individuals were analyzed. Because we could not find any previous studies to help us with effect sizes, we used the Cohen’s criterion, assuming a moderate effect size of 0.25 [21] and a group size of 6 to calculate 158 as the total sample size. Therefore, the total of 464 individuals in the present study is considered a sample size that meets the number required to assess gene–environment interaction in this study.

### Measurements

Age, sex, height, weight, waist circumference, and systolic and diastolic blood pressures (BPs) were assessed during the health checkups of all participants. BMI was calculated as current body weight (kg) divided by height (in m^2^). Hypertension was defined as a mean systolic blood pressure of ≥140 mm Hg or a mean diastolic blood pressure of ≥90 mm Hg at health checkups or a history or previous treatment of hypertension in the questionnaire. Participants with a BMI of ≥25 were defined as obese.

### Assessment of fatty liver

Hepatic steatosis was determined via B-mode ultrasonography performed by experienced hepatologists. Hepatic steatosis was defined as the presence of ≥1 of the following observations: increased hepatorenal contrast, liver brightness, deep attenuation, or vascular blurring [22].

### Nutrient assessment

The standardized methodology was used to evaluate nutritional intake based on the data obtained using the Japanese version of the BDHQ. This tool is a diet history questionnaire that assesses the frequency of consuming 58 selected food and beverage items during the previous month. The reproducibility and validity of BDHQ have been reported in a previous study [23].

We excluded all participants with extremely low or high EIs (<600 kcal/d, which is 50% of the required energy for the lowest physical activity category, or ≥4000 kcal/d, which is 1.5 times higher than the EI required for the highest physical activity category; *n* = 5).

### Alcohol consumption

BDHQ was used to assess daily alcohol consumption. It evaluates the amount and frequency of drinking Japanese sake, beer, wine, whiskey, and brandy.

### Blood sample collection and assays

Fasting blood samples were collected from each participant between 08:00 and 12:00 from the forearm vein after an overnight fast. The serum samples were delivered to Kanazawa University via a commercial laboratory (SRL Kanazawa Laboratory, Kanazawa, Japan). The sera were frozen and stored at −30 °C until further assay.

The HOMA-IR and the homeostasis model assessment of beta-cell function (HOMA-β), which are indices of insulin resistance and secretory capacity, respectively, were used [[24], [25], [26]]. A detailed calculation was performed using the following formula: HOMA-IR = [fasting insulin (μU/mL) × fasting glucose (mg/dL)]/405, and HOMA-β = [fasting insulin (μU/mL) × 360]/[fasting glucose (mg/dL) − 63].

Diabetes mellitus was defined as a HbA1c of ≥6.5% based on blood sampling or a history or under treatment of diabetes mellitus in the questionnaire.

Dyslipidemia was defined as a fasting TG concentration of ≥150 mg/dL, HDL cholesterol concentration of <40 mg/dL, Friedewald-estimated LDL cholesterol concentration of ≥140 mg/dL [27], or a history or previous treatment of dyslipidemia in the questionnaire.

### Genetic analyses

We extracted genomic DNA from the blood samples using a QIAamp DNA Blood Maxi Kit (Qiagen), according to the manufacturer’s instructions or consigning the company specializing in clinical laboratory testing (SRL Inc.). Genome-wide SNP genotyping was performed using Japonica Array v2 [28] (TOSHIBA Co., Ltd.). Details of the QC procedures for the genome-wide SNP genotype data obtained have been described in a previous study [29]. Briefly, QC filtering of SNPs and participants was based on sex identities between karyotypes and questionnaire findings, call rate, Hardy–Weinberg equilibrium test, inbreeding coefficient, cryptic relatedness, and population structure. In this study, we evaluated 31 previously reported NAFLD-associated SNPs that could be analyzed with Japonica Array. The genotypes of 31 NAFLD-associated SNPs [6,7,[30], [31], [32], [33], [34], [35], [36], [37], [38], [39], [40], [41], [42], [43], [44], [45], [46], [47]] for 464 unrelated participants (based on genome-wide$\hat{\pi }$ values) who passed the QC were extracted from the array data. In the QC step, the call rates of the 31 SNPs tested in this study were higher than 98%, and departures from the Hardy–Weinberg equilibrium were not observed in these SNPs (*P* > 0.03).

### Statistical analysis

Continuous variables were presented as means and SDs, and discrete variables were presented as frequencies and proportions. The differences between the baseline characteristics of patients with and without NAFLD were examined using Student’s or Welch’s t-test. The prevalence of diabetes mellitus and NAFLD in participants with the SNP genotypes were compared using Pearson’s chi-square test or Fisher’s exact test. Multiple logistic regression analysis adjusted for age, sex, and BMI was performed for the SNP that was significantly different in the chi-square analysis. One-way ANCOVA was performed to compare the means of nutrient intakes among participants with the TT, TC, and CC genotypes of rs2854116. ANCOVA was adjusted for covariates such as sex, BMI, and age. Two-way ANCOVA of NAFLD and rs2854116 genotypes was conducted to compare the mean nutrient intakes and examine the interactions between NAFLD and rs2854116 genotypes. Both the NAFLD and rs2854116 genotypes were used as between-subject variables. The same covariates shown above were used for adjustments. We conducted post-hoc tests with Bonferroni correction to examine the differences in the nutrient intakes between participants with and without NAFLD in each genotype. Then, multiple logistic regression analysis was performed via stratification with the rs2854116 genotypes. *P* values of <0.05 were considered statistically significant. All statistical analyses were conducted using the Statistical Package for the Social Sciences software version 25.0 (IBM).

## Results

### Participant characteristics

The mean age of the participants was 62.42 ± 11.33 y. Approximately 37.1% of them were men and 13.4% had diabetes mellitus. Participants with NAFLD were younger than those without it. Moreover, they had a lower HDL cholesterol concentration and higher BMI; waist circumference; body fat percentage; diastolic blood pressure; TG, glutamic oxaloacetic transaminase (GOT), glutamic pyruvic transaminase (GPT), gamma-glutamyl transpeptidase (γ-GTP), fasting plasma glucose (FPG), and HbA1c concentrations; and HOMA-IR and HOMA-β scores (Table 1). According to sex, female participants with NAFLD had a significantly higher HOMA-β score than those without it. Male participants with NAFLD had significantly higher GOT, γ-GTP, and HbA1c concentrations than those without it. Minor allele frequencies for 31 SNPs were similar to the previously reported allele frequencies of East Asians for dbSNP (Supplemental Table 1).

**TABLE 1:** Baseline characteristics of participants

	All (*n* = 464)^1^				Men (*n* = 172)			Women (*n* = 292)		
		Non-NAFLD (*n* = 301)	NAFLD (*n* = 163)	*P* value^2^	Non-NAFLD (*n* = 101)	NAFLD (*n* = 71)	*P* value	Non-NAFLD (*n* = 200)	NAFLD (*n* = 92)	*P* value
	Mean ± SD				Mean ± SD			Mean ± SD		
Age (y)	62.42 ± 11.33	64.16 ± 11.36	59.19 ± 10.58	<0.001	65.40 ± 10.91	59.72 ± 11.72	0.001	63.54 ± 11.56	58.78 ± 9.65	0.001
BMI (kg/m^2^)	23.25 ± 3.20	22.49 ± 2.98	24.65 ± 3.12	<0.001	23.25 ± 2.69	25.27 ± 2.76	<0.001	22.10 ± 3.05	24.17 ± 3.31	<0.001
WC (cm)	83.89 ± 8.97	82.04 ± 8.72	87.29 ± 8.43	<0.001	83.67 ± 8.52	89.03 ± 7.36	<0.001	81.23 ± 8.73	85.95 ± 8.98	<0.001
Body fat percentage (%)	30.65 ± 7.45	28.73 ± 7.30	32.37 ± 7.46	0.001	23.39 ± 5.13	27.22 ± 5.63	<0.001	32.72 ± 6.18	36.09 ± 6.34	<0.001
Systolic BP (mm Hg)	137.53 ± 18.93	137.49 ± 19.15	137.60 ± 18.59	0.955	141.30 ± 17.85	138.77 ± 16.65	0.350	135.57 ± 19.53	136.68 ± 20.01	0.653
Diastolic BP (mm Hg)	79.40 ± 11.24	77.92 ± 11.08	82.13 ± 11.04	<0.001	80.06 ± 11.23	84.86 ± 11.38	0.007	76.84 ± 10.87	80.02 ± 10.35	0.019
HDL-C (mg/dL)	63.92 ± 15.74	66.16 ± 15.84	59.80 ± 14.73	<0.001	59.94 ± 12.08	53.49 ± 12.65	0.001	69.31 ± 16.6	64.66 ± 14.42	0.021
TG (mg/dL)	113.02 ± 58.46	105.58 ± 57.88	126.72 ± 57.21	<0.001	115.89 ± 65.12	134.89 ± 53.95	0.045	100.35 ± 53.25	120.42 ± 59.12	0.004
GOT (IUL)	23.01 ± 8.13	22.36 ± 6.64	24.20 ± 10.25	0.040	22.77 ± 6.55	27.54 ± 13.29	0.006	22.16 ± 6.69	21.63 ± 5.99	0.521
GPT (IUL)	21.59 ± 13.61	18.68 ± 8.84	26.94 ± 18.42	<0.001	20.56 ± 9.40	34.94 ± 23.75	<0.001	17.72 ± 8.40	20.76 ± 9.05	0.005
γ-GTP (IUL)	34.14 ± 32.57	29.84 ± 26.04	42.06 ± 40.94	0.001	20.90 ± 30.80	59.04 ± 53.11	0.011	24.23 ± 21.22	28.95 ± 20.37	0.075
FPG (mg/dL)	95.08 ± 15.43	93.83 ± 14.90	97.32 ± 16.14	0.022	96.76 ± 15.22	100.14 ± 16.50	0.175	92.32 ± 14.55	95.13 ± 15.61	0.143
HbA1c (%)	5.94 ± 0.61	5.89 ± 0.60	6.03 ± 0.60	0.018	5.86 ± 0.46	6.09 ± 0.60	0.007	5.91 ± 0.66	5.98 ± 0.61	0.351
Diabetes mellitus (n, %)	62 (13.4)	32 (10.6)	30 (18.4)	0.019	14 (13.9)	16 (22.5)	0.140	18 (9.0)	14 (15.2)	0.114
HOMA-IR	1.27 ± 0.97	1.09 ± 0.72	1.59 ± 1.25	<0.001	1.13 ± 0.79	1.55 ± 0.91	0.002	1.07 ± 0.68	1.63 ± 1.47	0.001
HOMA-β (%)	65.79 ± 45.07	60.00 ± 36.43	76.27 ± 56.13	0.001	57.41 ± 44.56	69.33 ± 4.27	0.097	61.34 ± 31.45	81.71 ± 62.51	0.005

BP, blood pressure; FPG, fasting plasma glucose; GOT, glutamic oxaloacetic transaminase; GPT, glutamic pyruvic transaminase; γ-GTP, gamma-glutamyl transpeptidase; HOMA-β, homeostasis model assessment of insulin beta-cell function; NAFLD, nonalcoholic fatty liver disease; T-Cho, total cholesterol; WC, waist circumference.

1 All values are expressed as mean ± SD.

2 Student’s t-test or Welch’s t-test was used to compare all values except for diabetes. Pearson’s chi-square test was used to compare the prevalence of obesity, hypertension, dyslipidemia, and diabetes.

### Relationships between NAFLD and SNPs and their interactions with environmental factors

Among the 31 SNPs, only polymorphism T-455C in the *APOC3* gene (rs2854116) was significantly associated with fatty liver disease (*P* = 0.003; Table 2). However, multiple logistic regression analysis adjusted for age, sex, and BMI using the TT genotype as a reference category showed a higher NAFLD risk in heterozygotes (OR: 1.656; 95% CI: 1.019–2.691; *P* = 0.042). There were no significant differences in NAFLD prevalence in the dominant or recessive model. The C allele frequencies were 0.45 and 0.46 in participants with and without NAFLD, respectively. T-455C polymorphism was not associated with BMI; waist circumference; body fat percentage; TG, HDL cholesterol, total cholesterol, γ-GTP, fasting glucose, and HbA1c concentrations; and HOMA-IR and HOMA-β scores. However, GOT concentrations (*P* = 0.082) and GPT concentrations (*P* = 0.064) tended to differ between the participants with and without NAFLD (Supplemental Table 2).

**TABLE 2:** Association tests of the SNPs in participants with NAFLD and non-NAFLD^1^

SNP ID	Nearby gene	Major allele (X)	Minor allele (x)	NAFLD	non-NAFLD	*P* value^1^
				*n* (XX/Xx/xx)	*n* (XX/Xx/xx)	
rs1801133	*MTHFR*	G	A	65/79/19	116/138/46	0.551
rs1137101	*LEPR*	G	A	125/34/4	225/72/4	0.532
rs2499604	*ZP4*	T	C	40/81/42	77/156/68	0.745
rs1260326	*GCKR*	T	C	56/81/25	80/165/56	0.187
rs780094	*GCKR*	T	C	54/80/29	88/153/60	0.657
rs4374383	*MERTK*	A	G	103/55/5	183/105/13	0.754
rs16944	*IL-1*	G	A	47/82/33	88/152/61	0.999
rs1801278	*IRS-1*	C	T	152/11/0	279/19/0	0.877
rs1801282	*PPARγ*	C	G	150/13/0	271/29/1	0.749
rs3772622	*AGTR1*	T	C	54/76/33	100/147/54	0.816
rs2710833	*DDX60L*	C	T	126/36/1	223/73/4	0.718
rs2569190	*CD14*	A	G	53/72/38	96/157/48	0.107
rs1042714	*ADRB2*	C	G	140/20/3	251/48/2	0.301
rs4880	*SOD2*	A	G	124/37/2	232/67/2	0.772
rs4994	*ADRB3*	A	G	105/50/8	196/98/7	0.316
rs1227756	*COL13A1*	G	A	85/64/14	170/107/22	0.615
rs7903146	*TCF7L2*	C	T	143/19/0	268/31/0	0.654
rs6591182	*LTBP3*	T	G	50/79/34	85/150/65	0.869
rs2854116	*APOC3*	T	C	42/95/26	98/127/76	0.003
rs7946	*PEMT*	C	T	76/69/18	153/125/23	0.411
rs1421201	No gene	A	G	56/74/33	114/130/56	0.733
rs2228603	*NCAN*	C	T	138/25/0	268/30/2	0.142
rs58542926	*TM6SF2*	C	T	133/30/0	256/43/1	0.463
rs429358	*APOE*	T	C	132/30/1	230/66/3	0.604
rs7412	*APOE*	C	T	146/17/0	269/32/0	0.946
rs641738	*TMC4, MBOAT7*	C	T	101/53/8	192/90/16	0.851
rs738409	*PNPLA3*	C	G	46/81/36	82/159/59	0.758
rs2896019	*PNPLA3*	T	G	47/83/33	90/152/59	0.967
rs3761472	*SAMM50*	A	G	54/79/30	96/147/57	0.967
rs2143571	*SAMM50*	G	A	52/77/34	95/147/59	0.931
rs6006611	*PARVB*	G	A	52/74/37	97/142/61	0.831

APOC3, apolipoprotein C3; NAFLD, nonalcoholic fatty liver disease; SNPs, single nucleotide polymorphisms.

1 Pearson’s chi-square or Fisher’s exact test was performed to compare the prevalence of NAFLD among SNP genotypes.

Next, we examined physical and biochemical data that interact with rs2854116 and NAFLD. Neither physical characteristics (such as BMI, waist circumference, and body fat percentage) nor biochemical factors (including TG, HDL cholesterol, total cholesterol, GOT, GPT, γ-GTP, fasting glucose, and HbA1c concentrations and HOMA-IR and HOMA-β scores) showed any interaction between rs2854116 and NAFLD. After that, we examined dietary data such as the intake of energy, protein, fat, animal fat, vegetable fat, carbohydrates, sucrose, SFAs, MUFAs, PUFAs, cholesterol, n-3 FAs, n-6 FAs, and alcohol. Table 3 shows dietary variables between the rs2854116 genotypes. No main effects of nutrient intakes on NAFLD or *APOC3* genotypes were observed. However, significant interactions were observed between NAFLD and *APOC3* genotypes and the intake of fat (*P* = 0.006), vegetable fat (*P* = 0.017), MUFAs (*P* = 0.002), PUFAs (*P* = 0.015), cholesterol (*P* = 0.031), n-3 FAs (*P* = 0.038), and n-6 FAs (*P* = 0.025). In other words, participants with NAFLD who have TT genotype (rs2854116) have a higher fat intake than participants without NAFLD. The intake of carbohydrates (*P* = 0.060) and SFAs (*P* = 0.087) had a trend for interaction between NAFLD and the *APOC3* genotypes. Multiple comparisons with the Bonferroni method showed a significant difference in fat intake between the participants with TT genotype and NAFLD and those without it (*P* = 0.001). The NAFLD group had a significantly higher fat intake and remarkably lower carbohydrate intake (*P* = 0.049) than the non-NAFLD group (Table 3). Similar findings were observed for vegetable fat (*P* = 0.002), MUFAs (*P* < 0.001), PUFAs (*P* = 0.008), cholesterol (*P* = 0.005), and n-6 fatty acids (*P* = 0.008; Table 3). In the non-NAFLD group, participants with the CC genotype had a higher fat intake than those with the TT genotype (*P* = 0.011). Similar results were noted in SFAs (*P* = 0.023), MUFAs (*P* = 0.020), and cholesterol (*P* = 0.004; Supplemental Table 3).

**TABLE 3:** Dietary characteristics of participants by the genotype of *APOC3* T-455C (rs2854116)

Nutrients	NAFLD	Total (*n* = 464)	TT (*n* = 140)	TC (*n* = 222)	CC (*n* = 102)	*P* value for NAFLD^1^	*P* value for rs2854116^1^	*P* value for interaction^1^	*P* value for TT^2^	*P* value for TC^2^	*P* value for CC^2^
Energy (kcal)	−	1750.2 ± 573.2	1724.6 ± 583.5	1759.4 ± 584.1	1767.8 ± 547.1	0.744	0.655	0.363			
	+	1806.9 ± 599.5	1868.2 ± 568.3	1825.3 ± 604.7	1640.4 ± 622.8						
Protein (% of energy)	−	15.7 ± 3.2	15.2 ± 3.0	15.7 ± 3.2	16.3 ± 3.5	0.868	0.450	0.193			
	+	15.4 ± 3.1	15.3 ± 3.1	15.6 ± 3.1	14.9 ± 3.0						
Fat (% of energy)	−	24.8 ± 5.6	23.7 ± 5.8	24.8 ± 5.5	26.3 ± 5.1	0.146	0.803	0.006	0.001	0.137	0.163
	+	26.2 ± 6.1	27.4 ± 7.1	25.9 ± 5.8	25.1 ± 5.2						
Animal fat (% of energy)	−	11.7 ± 4.0	11.1 ± 4.0	11.6 ± 3.9	12.8 ± 3.9	0.547	0.370	0.140			
	+	12.2 ± 4.0	12.6 ± 4.2	12.0 ± 3.9	12.3 ± 4.0						
Vegetable fat (% of energy)	−	13.1 ± 3.3	12.7 ± 3.4	13.2 ± 3.5	13.5 ± 3.0	0.100	0.420	0.017	0.002	0.085	0.261
	+	14.0 ± 3.9	14.8 ± 4.4	14.0 ± 3.8	12.9 ± 3.2						
CHO (% of energy)	−	56.6 ± 7.6	57.4 ± 7.8	56.8 ± 7.6	55.2 ± 7.2	0.578	0.864	0.060	0.049	0.312	0.164
	+	55.5 ± 7.9	54.3 ± 9.0	55.6 ± 7.3	57.1 ± 8.3						
Sucrose (% of energy)	−	2.7 ± 1.9	2.6 ± 2.0	2.7 ± 1.9	2.7 ± 1.9	0.075	0.762	0.624			
	+	2.9 ± 1.8	3.1 ± 2.0	2.9 ± 1.8	2.7 ± 1.6						
SFAs (% of energy)	−	6.6 ± 1.8	6.3 ± 1.8	6.5 ± 1.7	7.0 ± 1.9	0.190	0.615	0.087	0.020	0.124	0.403
	+	6.9 ± 1.8	7.1 ± 1.9	6.9 ± 1.8	6.9 ± 1.7						
MUFAs (% of energy)	−	8.7 ± 2.2	8.3 ± 2.2	8.7 ± 2.2	9.2 ± 2.0	0.096	0.626	0.002	<0.001	0.120	0.151
	+	9.3 ± 2.4	10.0 ± 2.8	9.2 ± 2.4	8.8 ± 2.0						
PUFAs (% of energy)	−	6.2 ± 1.4	5.9 ± 1.4	6.2 ± 1.5	6.4 ± 1.2	0.465	0.875	0.015	0.008	0.501	0.135
	+	6.4 ± 1.6	6.7 ± 2.0	6.3 ± 1.4	6.1 ± 1.6						
Cholesterol (mg/1000 kcal)	−	201.5 ± 75.8	185.4 ± 73.3	201.1 ± 72.7	222.7 ± 79.6	0.175	0.762	0.031	0.005	0.195	0.291
	+	212.0 ± 68.6	219.9 ± 78.9	211.2 ± 63.6	201.9 ± 70.1						
n-3 FAs (% of energy)	−	1.3 ± 0.4	1.3 ± 0.4	1.4 ± 0.4	1.4 ± 0.4	0.829	0.978	0.038	0.060	0.693	0.107
	+	1.3 ± 0.4	1.4 ± 0.5	1.3 ± 0.4	1.3 ± 0.5						
n-6 FAs (% of energy)	−	4.8 ± 1.2	4.6 ± 1.1	4.8 ± 1.2	5.0 ± 1.0	0.319	0.821	0.025	0.008	0.322	0.200
	+	5.0 ± 1.3	5.3 ± 1.6	5.0 ± 1.1	4.8 ± 1.2						
Alcohol (g/d)	−	3.9 ± 7.2	5.6 ± 8.3	3.3 ± 6.6	2.6 ± 6.2	0.332	0.449	0.198			
	+	4.0 ± 7.3	4.0 ± 7.9	4.0 ± 7.4	3.8 ± 6.6						

APOC3, apolipoprotein C3; NAFLD, nonalcoholic fatty liver disease.

1 Two-way ANCOVA between NAFLD and rs2854116 genotypes adjusted by sex, BMI, and age. ^2^ Bonferroni post-hoc test.

When stratified according to the rs2854116 genotype, multiple logistic regression analysis showed that participants with NAFLD who presented with the TT genotype had higher intakes of fat (OR: 1.102; 95% CI: 1.026–1.183; *P* = 0.007), vegetable fat (OR: 1.171; 95% CI: 1.046–1.311; *P* = 0.006), SFAs (OR: 1.264; 95% CI: 1.007–1.588; *P* = 0.043), MUFAs (OR: 1.322; 95% CI: 1.104–1.582; *P* = 0.002), PUFAs (OR: 1.334; 95% CI: 1.030–1.728; *P* = 0.029), and n-6 fatty acids (OR: 1.413; 95% CI: 1.029–1.940; *P* = 0.033) than those without it. Participants with NAFLD who presented with the TT genotype were more likely to have a higher intake of sucrose, cholesterol, and n-3 fatty acids than those without NAFLD (Supplemental Table 4).

**FIGURE 2:**
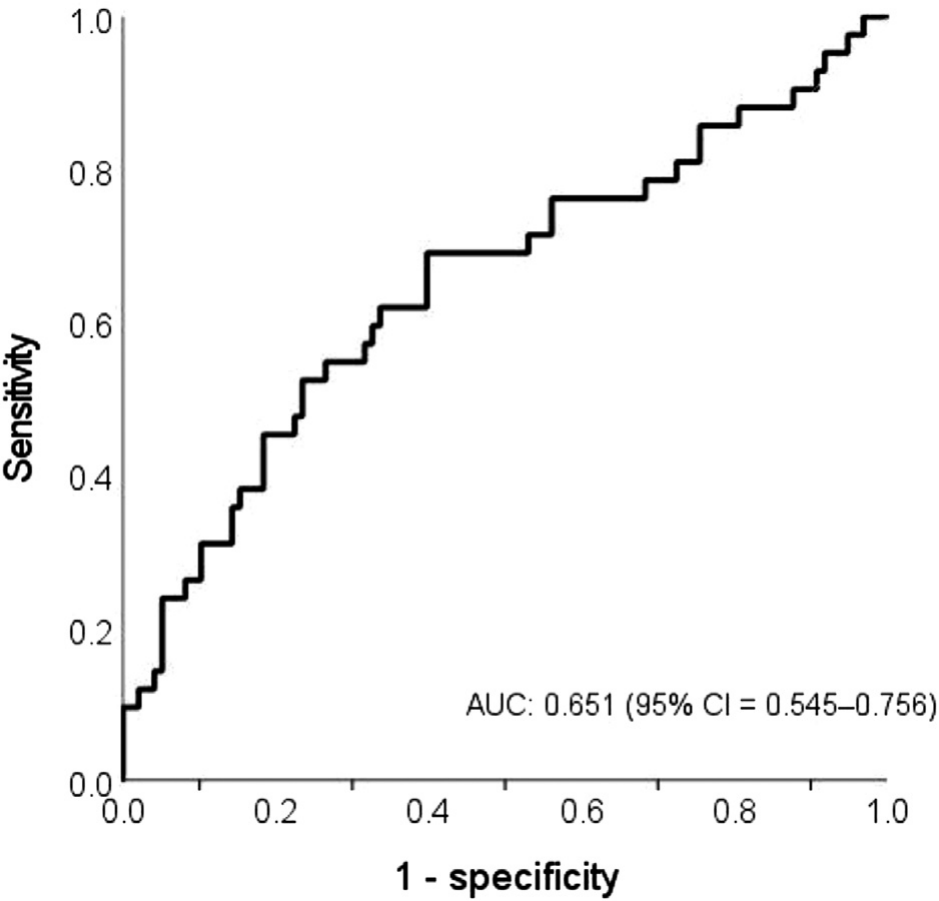
ROC curves for lipid intake in participants with TT genotype as a predictor of risk of NAFLD. The area under the ROC curve was 0.651 (95% CI: 0.545–0.756; *P* = 0.005). The optimum cut-off value for lipid intake with the Youden index was 25.4% (sensitivity: 0.690; specificity: 0.602). NAFLD, nonalcoholic fatty liver disease; ROC, receiver operating characteristic.

Next, we calculated a cut-off concentration of fat intake, which elevates fatty liver risk in participants with the TT genotype. Regarding the cut-off concentrations of fat intake, we have listed the nutrient intake status in each genotype of the *APOC3* gene (rs2854116) in Table 3. We used the receiver operating characteristic (ROC) curves to identify the AUC and the cut-off concentrations with the Youden index. For participants with the TT genotype, the optimal cut-off concentrations were determined to be 24.5% of the fat intake with the highest Youden index (sensitivity: 0.690; specificity: 0.602). AUC for fat intake was 0.651 (95% CI: 0.545–0.756; *P* = 0.005; Figure 2).

## Discussion

Unexpectedly, most of the previously recognized NAFLD-associated genes, including *PNPLA3*, were not simply related to fatty liver in the present study. This is the general population–based cohort but not the disease cohort. Although fibrosis was not assessed in the present study, the prevalence of NASH in general population health examinations has been reported to be as low as a few percent [2]. The genome-wide association analysis of the Japanese population has shown an association between *PNPLA3* mutations and NAFLD but reported no association with Matteoni classification type 1 to type 3 [5]. Therefore, *PNPLA3* genotypes may not be involved in simple fatty liver and relatively mild NASH without fibrosis. A glucokinase regulator gene (*GCKR*) have also been reported to be significantly associated with NAFLD [33,6]. However, this study did not show an association.

Nevertheless, to the best of our knowledge, this is the first study to demonstrate the effect of genetic predisposition on the association between nutrient intakes and NAFLD at the general population level. The *APOC3* T-455C polymorphism (rs2854116) interacts with the correlation between fat intake and NAFLD. The participants with TT genotype (rs2854116) and NAFLD show a higher fat intake than those without NAFLD. We did not observe the interaction of fat intake with NAFLD in the TC genotype. In the present study, fat and carbohydrate intakes traded off each other. Participants who consumed high fat consumed low carbohydrates. Indeed, the intake of carbohydrates had a trend for interaction between NAFLD and the *APOC3* genotypes. Participants with the TT genotype who took higher fat or lower carbohydrate were associated with fatty liver. These findings indicate that polymorphism T-455C in the *APOC3* gene (rs2854116) interacts with the association between nutrient intake and NAFLD, as observed in *PNPLA3* gene polymorphism [17].

To date, >100 SNPs have been found to be associated with NAFLD [48,49]. In this study, we evaluated 31 previously reported NAFLD-associated SNPs available in our cohort data. None of the SNPs that are significantly associated with NAFLD could be detected except for *APOC3* T-455C. Fatty liver was more common in participants with heterozygote mutations of the polymorphism T-455C in the *APOC3* gene (rs2854116) than in those with homozygotes (TT and CC). A similar example of heterozygosity associated with disease development was reported in the association between aldehyde dehydrogenase 2 (ALDH2) gene mutation and esophageal cancer [50]. *ALDH2* gene mutation (rs671) is an SNP frequently found in East Asians. Individuals with homozygotes for the variant allele ∗2 of *ALDH2* cannot metabolize acetaldehyde. In those individuals with ∗1/∗2, the blood acetaldehyde concentration after alcohol consumption is 6 times higher than in those with ∗1/∗1. Risk of esophageal cancer is significantly increased in ∗2/∗2 individuals if they drink, but they usually drink little. Meta-analyses show that ∗1/∗2 are at an increased risk of carcinogenesis, which is even higher for heavy drinkers. In this instance, heterozygosity is more associated with disease development than homozygosity.

The contribution of LPL to liver steatosis seems complex. LPL is required for fat intake from the diet in the liver by hydrolyzing chylomicrons to fatty acids and chylomicron remnants. Inhibition of LPL predisposes to hypertriglyceridemia because of increased chylomicron remnants. Increased circulating chylomicron remnants are preferentially taken up by the liver [51], leading to nonalcoholic fatty liver disease and hepatic insulin resistance. A recent study showed that *APOC3* transgenic mice exhibit hypertriglyceridemia [52]. However, the severity of fatty liver disease in these mice did not differ from that in wild-type mice even if they were fed a high-fructose or high-fat diet [52]. Other studies have reported that *APOC3* variants are not associated with NAFLD in humans [53,54]. By contrast, in nonobese Asian Indian men, carriers of the T allele of *APOC3* C-482T (rs2854117), C allele of *APOC3* T-455C (rs2854116), or both are associated with elevated APOC3 concentrations, reduced plasma TG clearance, hypertriglyceridemia, and a higher prevalence of NAFLD than those with the homozygotes of the opposite alleles [7]. The findings were inconsistent, suggesting that other factors interact with the genotype, thereby increasing risk of NAFLD.

In this study, *APOC3* T-455C-mediated inactivation of LPL was not significantly associated with fatty liver disease. However, nutrition intake interacted with the *APOC3* T-455C genotype and fatty liver. Only participants with fatty liver in subsamples with the TT genotype for rs2854116 have a higher fat intake. Based on the cut-off concentration estimated by the ROC curve, it may be helpful to recommend the participants with the TT genotype keep their lipid intake below 24.5% of energy to avoid fatty liver. We propose 2 hypotheses to interpret this finding. First, a high-fat diet is more likely to cause a fatty liver than high carbohydrate intake in people who present with the TT genotype of this SNP. Second, the TT genotype of rs2854116 might cause patients with a fatty liver to prefer fat over carbohydrates. These hypotheses should be investigated in the future.

Macronutrient balance affects lipid metabolism. A fructose-enriched diet elevates serum APOC3 concentrations in men [10]. Conversely, a 2-wk intervention with an isocaloric low-carbohydrate diet (<30 g/d) with high fat and protein contents reduced plasma APOC3 concentrations in patients with obesity who presented with NAFLD [55]. Another study has reported that the intake of saturated fat elevates plasma APOC3 concentrations, whereas that of monounsaturated and polyunsaturated fat has been associated with decreased plasma APOC3 concentrations [11]. In addition, n-3 fatty acids have also been reported to reduce plasma APOC3 concentrations [13]. These findings suggest that a high-fat diet, specifically monounsaturated and polyunsaturated fats, may degrade circulating APOC3 concentrations. However, in the present study, consumption of higher amounts of MUFAs and PUFAs among participants with the TT genotype and the total fat intake were associated with fatty liver. SFAs also showed a trend of interaction. Therefore, it is challenging to specify particular FAs involved in the TT genotype–associated NAFLD. Unfortunately, because we did not assess the blood APOC3 concentrations in the present study, it is unclear what nutrition and genotypes contribute to APOC3 concentrations, which should be investigated in the future.

The sources of liver fat as described in a previous report are as follows: 59% from visceral fat flux into the liver, 26% from de novo lipogenesis, and 15% from the diet. However, individual differences were observed [56]. Carbohydrates and insulin enhance de novo lipogenesis by upregulating master transcription factors ChREBP and SREBP-1c, respectively [57]. ChREBP regulates more than half of de novo lipogenesis [58]. Ketone bodies inhibit the nuclear localization of ChREBP [59]. Humans with the TT genotype of the *APOC3* T-455C polymorphism (rs2854116) have lower APOC3 concentrations than those with the C allele [7]. Lower APOC3 concentrations may elevate LPL activity, lipolysis, FFAs, and ketone bodies. Elevated ketone bodies may suppress ChREBP, resulting in relatively less contribution of carbohydrate-induced de novo lipogenesis to hepatic triacylglycerol synthesis. It is possible that dietary fat has more effect on liver steatosis than carbohydrate-derived fat in humans with the TT genotype of the *APOC3* T-455C polymorphism (rs2854116). However, we did not assess the blood APOC3 or ketone concentrations in the present study. Thus, further studies should be performed.

Our study has several limitations. First, the study is cross-sectional and does not allow us to know the causal role of dietary fat intake in fatty liver development among genotypes. Second, the subjects were Japanese individuals aged ≥40 y. A large proportion of them were women because we excluded moderate drinkers. These were addressed by controlling for age and gender in the analysis. Third, out of >100 candidate NAFLD-related SNPs, we analyzed 31 SNPs available in Japonica Array v2. Additional studies with larger samples, including more men, adults aged 20–40 y, and other ethnic groups, would be desirable to confirm our conclusion in the future.

Whether dietary carbohydrates or fats contribute to NAFLD development remains controversial. Based on our results, genetic predisposition to energy-related genes might interact with the association between nutrient intake and NAFLD development, which could help us with personalized nutritional guidance. Nevertheless, whole genome sequencing is not currently available for all people. Advances in nutrigenomics are expected to identify biomarkers for genetic information affecting nutrient metabolism.

In conclusion, polymorphism T-455C in the *APOC3* gene (rs2854116) and fat intake are associated with risk of NAFLD in Japanese adults. Participants with fatty liver who presented with the TT genotype of rs2854116 have a higher fat intake. Such nutrigenetic interaction could deepen our understanding of the NAFLD pathology and contribute to the current practice of healthcare professionals based on accurate knowledge. In addition, the current study showed that the correlation between genetic factors and nutrition intake should be considered in personalized nutritional interventions against NAFLD.

## Funding

The authors reported no funding received for this study.

## Author disclosures

RY, YT, HT, TK, TS, KH, KS, YK, TTanaka, HG, YN, TY, SK, AT, HN, and TTakamura, no conflicts of interest.

## Data Availability

The datasets presented in this article are not readily available due to several ongoing studies using the datasets in this study. Requests to access the datasets should be directed to ttakamura@med.kanazawa-u.ac.jp.

## Appendix A Supplementary data

Multimedia component1 Multimedia component1
